# 2,2′-*o*-Phenyl­enediacetonitrile

**DOI:** 10.1107/S1600536809041506

**Published:** 2009-10-17

**Authors:** Yang Li, Guoxiong Hua, Alexandra M. Z Slawin, J. Derek Woollins

**Affiliations:** a Department of Chemistry, University of St Andrews, St Andrews KY16 9ST, Scotland

## Abstract

In the title compound, NCCH_2_C_6_H_4_CH_2_CN, the bond lengths and angles are within normal ranges. The benzene ring makes dihedral angles of 4.94 (8) and 77.04 (8)° with the C—C—C—N mean planes. Weak non-conventional C—H⋯N hydrogen bonds are effective in the stabilization of the crystal structure. The weak C—H⋯N contacts form anti­parallel chains running in the *a* + *c* direction, and ring systems with two N-atom acceptors and four H-atom donors.

## Related literature

For reactions of Woollins’ Reagent see: Gray *et al.* (2005 ▶); Hua *et al.* (2006 ▶, 2009 ▶); Hua & Woollins (2009 ▶). For bond-length data, see: Allen *et al.* (1987 ▶).
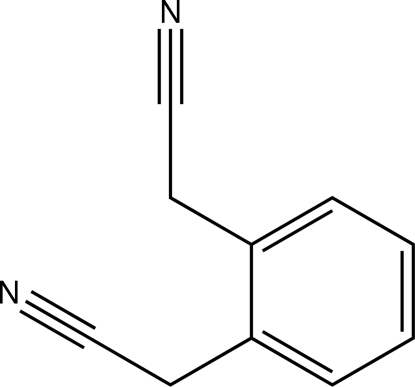


## Experimental

### 

#### Crystal data

C_10_H_8_N_2_
*M* *_r_* = 156.18
Monoclinic, 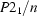

*a* = 8.3882 (18) Å
*b* = 8.1605 (15) Å
*c* = 11.993 (2) Å
β = 101.890 (6)°
*V* = 803.4 (3) Å^3^
*Z* = 4
Mo *K*α radiation
μ = 0.08 mm^−1^
*T* = 93 K
0.30 × 0.25 × 0.15 mm

#### Data collection

Rigaku Mercury CCD diffractometer
Absorption correction: multi-scan (*CrystalClear*; Rigaku, 2004 ▶) *T*_min_ = 0.977, *T*_max_ = 0.988
5271 measured reflections
1660 independent reflections
1330 reflections with *I* > 2σ(*I*)
*R*_int_ = 0.031

#### Refinement

*R*[*F* ^2^ > 2σ(*F* ^2^)] = 0.051
*wR*(*F* ^2^) = 0.133
*S* = 1.09
1660 reflections
109 parameters
H-atom parameters constrained
Δρ_max_ = 0.28 e Å^−3^
Δρ_min_ = −0.21 e Å^−3^

### 

Data collection: *CrystalClear* (Rigaku, 2004 ▶); cell refinement: *CrystalClear*; data reduction: *CrystalClear*; program(s) used to solve structure: *SHELXS97* (Sheldrick, 2008 ▶); program(s) used to refine structure: *SHELXL97* (Sheldrick, 2008 ▶); molecular graphics: *PLATON* (Spek, 2009 ▶); software used to prepare material for publication: *SHELXL97*.

## Supplementary Material

Crystal structure: contains datablocks I, global. DOI: 10.1107/S1600536809041506/si2203sup1.cif

Structure factors: contains datablocks I. DOI: 10.1107/S1600536809041506/si2203Isup2.hkl

Additional supplementary materials:  crystallographic information; 3D view; checkCIF report

## Figures and Tables

**Table 1:** Hydrogen-bond geometry (Å, °)

*D*—H⋯*A*	*D*—H	H⋯*A*	*D*⋯*A*	*D*—H⋯*A*
C9—H9*A*⋯N1^i^	0.99	2.57	3.5605 (18)	176
C9—H9*B*⋯N1^ii^	0.99	2.56	3.5210 (17)	165

Symmetry codes: (i) 
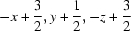
; (ii) 
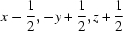
.

**Fig. 1.**
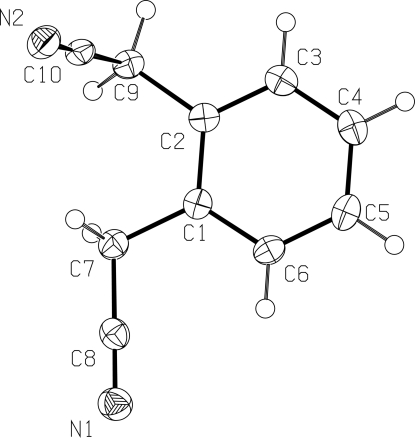
The molecular structure of the title compound with displacement ellipsoids drawn at the 50% probability level.

**Fig. 2.**
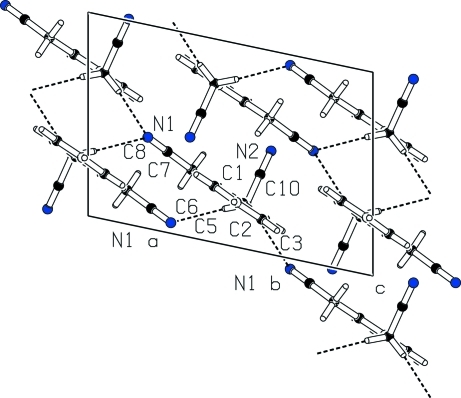
View along the b direction of the crystal packing of the title compound with non-conventional hydrogen bonding shown as dashed lines.

## supplementary crystallographic information

### Comment

Recently, we have continued our studies exploring the reactivity of Woollins reagent towards different organic substituents (Gray *et al.* 2005, Hua *et al.* 2006 and 2009; Hua & Woollins 2009). Thereby 2,2'-(1,2-phenylene)diacetonitrile represents one of the starting materials. Single crystals of 2,5-dihydroxybenzaldehyde for X-ray crystallographic analysis were obtained by recrystallization from dichloromethane-hexane solution.

The bond lengths (Allen *et al.*, 1987) and angles are within normal ranges.The benzene ring makes the dihedral angles of 4.94 (8) and 77.04 (8)° with the mean planes of C1—C7—C8—N1 and C2—C9—C10—N2 respectively. The antiparallel chains running in *a*+*c*direction are generated through the weak C—H···N contacts, glide plane and inversinon symmetry operations[see Fig. 2 and Table 1]. Inversion symmetry forms also C—H···N ring systems consisting of two N acceptors and four H atom donors,where the centroid-centroid distance between the inversion-related benzene ring planes is 3.6809 (10) Å, the perpendicular plane to plane distance is 3.364 Å, and the slippage between the planes is 1.495 Å.

### Experimental

Commercially available 2,2'-(1,2-phenylene)diacetonitrile was recrystallized from dichloromethane-hexane.

### Refinement

H atoms were positioned geometrically (C—H = 0.93–0.97 Å) and were treated as riding on their parent C atoms with *U*_iso_(H)= 1.2–1.5Ueq(C).

### Crystal data

**Table:**

C_10_H_8_N_2_	*F*(000) = 328
*M**_r_* = 156.18	*D*_x_ = 1.291 Mg m^−^^3^
Monoclinic, *P*2_1_/*n*	Mo *K*α radiation, λ = 0.71073 Å
*a* = 8.3882 (18) Å	Cell parameters from 2633 reflections
*b* = 8.1605 (15) Å	θ = 2.7–28.3°
*c* = 11.993 (2) Å	µ = 0.08 mm^−^^1^
β = 101.890 (6)°	*T* = 93 K
*V* = 803.4 (3) Å^3^	Block, colorless
*Z* = 4	0.30 × 0.25 × 0.15 mm

### Data collection

**Table:**

Rigaku Mercury CCD diffractometer	1660 independent reflections
Radiation source: rotating anode	1330 reflections with *I* > 2σ(*I*)
confocal	*R*_int_ = 0.031
Detector resolution: 0.83 pixels mm^-1^	θ_max_ = 28.7°, θ_min_ = 2.7°
ω scans	*h* = −11→10
Absorption correction: multi-scan (*CrystalClear*; Rigaku, 2004)	*k* = −9→10
*T*_min_ = 0.977, *T*_max_ = 0.988	*l* = −13→15
5271 measured reflections	

### Refinement

**Table:**

Refinement on *F*^2^	Primary atom site location: structure-invariant direct methods
Least-squares matrix: full	Secondary atom site location: difference Fourier map
*R*[*F*^2^ > 2σ(*F*^2^)] = 0.051	Hydrogen site location: inferred from neighbouring sites
*wR*(*F*^2^) = 0.133	H-atom parameters constrained
*S* = 1.09	*w* = 1/[σ^2^(*F*_o_^2^) + (0.0698*P*)^2^ + 0.0077*P*] where *P* = (*F*_o_^2^ + 2*F*_c_^2^)/3
1660 reflections	(Δ/σ)_max_ = 0.001
109 parameters	Δρ_max_ = 0.28 e Å^−^^3^
0 restraints	Δρ_min_ = −0.21 e Å^−^^3^

### Special details

**Table:**

Geometry. All e.s.d.'s (except the e.s.d. in the dihedral angle between two l.s. planes) are estimated using the full covariance matrix. The cell e.s.d.'s are taken into account individually in the estimation of e.s.d.'s in distances, angles and torsion angles; correlations between e.s.d.'s in cell parameters are only used when they are defined by crystal symmetry. An approximate (isotropic) treatment of cell e.s.d.'s is used for estimating e.s.d.'s involving l.s. planes.
Refinement. Refinement of *F*^2^ against ALL reflections. The weighted *R*-factor *wR* and goodness of fit *S* are based on *F*^2^, conventional *R*-factors *R* are based on *F*, with *F* set to zero for negative *F*^2^. The threshold expression of *F*^2^ > σ(*F*^2^) is used only for calculating *R*-factors(gt) *etc*. and is not relevant to the choice of reflections for refinement. *R*-factors based on *F*^2^ are statistically about twice as large as those based on *F*, and *R*- factors based on ALL data will be even larger.

### Fractional atomic coordinates and isotropic or equivalent isotropic displacement parameters (Å^2^)

**Table:**

	*x*	*y*	*z*	*U*_iso_*/*U*_eq_	
C1	0.77845 (13)	0.05887 (16)	0.95174 (10)	0.0215 (3)	
C2	0.72184 (13)	0.13136 (16)	1.04228 (10)	0.0218 (3)	
C7	0.83384 (14)	0.16768 (16)	0.86390 (10)	0.0257 (3)	
H7A	0.7404	0.2350	0.8253	0.031*	
H7B	0.9191	0.2433	0.9038	0.031*	
C3	0.66532 (15)	0.03264 (16)	1.12009 (11)	0.0250 (3)	
H3	0.6273	0.0817	1.1816	0.030*	
C8	0.89813 (14)	0.07728 (16)	0.77779 (10)	0.0252 (3)	
N1	0.94789 (13)	0.00673 (14)	0.70957 (10)	0.0313 (3)	
C10	0.88145 (15)	0.38338 (15)	1.10361 (10)	0.0248 (4)	
C9	0.71906 (14)	0.31597 (16)	1.05579 (11)	0.0249 (3)	
H9A	0.6768	0.3666	0.9805	0.030*	
H9B	0.6438	0.3448	1.1064	0.030*	
C5	0.71958 (14)	−0.20835 (16)	1.02028 (11)	0.0278 (4)	
H5	0.7192	−0.3242	1.0126	0.033*	
C6	0.77646 (14)	−0.11076 (16)	0.94203 (11)	0.0248 (4)	
H6	0.8146	−0.1607	0.8809	0.030*	
N2	1.00792 (13)	0.43446 (14)	1.14226 (9)	0.0318 (3)	
C4	0.66348 (14)	−0.13701 (17)	1.10929 (11)	0.0271 (3)	
H4	0.6239	−0.2034	1.1628	0.033*	

### Atomic displacement parameters (Å^2^)

**Table:**

	*U*^11^	*U*^22^	*U*^33^	*U*^12^	*U*^13^	*U*^23^
C1	0.0169 (6)	0.0226 (7)	0.0238 (7)	0.0002 (4)	0.0009 (5)	0.0007 (5)
C2	0.0176 (6)	0.0247 (7)	0.0221 (7)	0.0023 (5)	0.0013 (5)	−0.0002 (5)
C7	0.0267 (7)	0.0232 (7)	0.0274 (7)	0.0013 (5)	0.0062 (5)	0.0001 (6)
C3	0.0207 (7)	0.0311 (8)	0.0220 (7)	−0.0002 (5)	0.0019 (5)	−0.0002 (6)
C8	0.0217 (7)	0.0285 (7)	0.0252 (7)	−0.0031 (5)	0.0043 (5)	0.0008 (6)
N1	0.0292 (7)	0.0337 (8)	0.0319 (7)	−0.0012 (5)	0.0085 (5)	−0.0019 (5)
C10	0.0321 (7)	0.0198 (7)	0.0243 (7)	0.0044 (5)	0.0097 (6)	−0.0012 (5)
C9	0.0256 (7)	0.0240 (8)	0.0250 (7)	0.0021 (5)	0.0049 (5)	−0.0018 (6)
C5	0.0257 (7)	0.0210 (7)	0.0342 (8)	−0.0006 (5)	0.0007 (6)	0.0018 (6)
C6	0.0241 (7)	0.0235 (8)	0.0263 (7)	0.0027 (5)	0.0040 (5)	−0.0023 (6)
N2	0.0325 (7)	0.0272 (7)	0.0359 (7)	−0.0019 (5)	0.0071 (5)	−0.0044 (5)
C4	0.0234 (7)	0.0314 (8)	0.0253 (7)	−0.0034 (5)	0.0022 (5)	0.0054 (6)

### Geometric parameters (Å, °)

**Table:**

C1—C6	1.3890 (19)	C8—N1	1.1471 (15)
C1—C2	1.4025 (16)	C10—N2	1.1452 (15)
C1—C7	1.5218 (18)	C10—C9	1.4714 (17)
C2—C3	1.3882 (18)	C9—H9A	0.9900
C2—C9	1.5159 (18)	C9—H9B	0.9900
C7—C8	1.4599 (17)	C5—C4	1.3809 (18)
C7—H7A	0.9900	C5—C6	1.3882 (17)
C7—H7B	0.9900	C5—H5	0.9500
C3—C4	1.390 (2)	C6—H6	0.9500
C3—H3	0.9500	C4—H4	0.9500
			
C6—C1—C2	119.02 (11)	N2—C10—C9	178.97 (14)
C6—C1—C7	121.58 (11)	C10—C9—C2	112.32 (10)
C2—C1—C7	119.36 (12)	C10—C9—H9A	109.1
C3—C2—C1	119.52 (13)	C2—C9—H9A	109.1
C3—C2—C9	119.34 (11)	C10—C9—H9B	109.1
C1—C2—C9	121.14 (11)	C2—C9—H9B	109.1
C8—C7—C1	113.88 (11)	H9A—C9—H9B	107.9
C8—C7—H7A	108.8	C4—C5—C6	120.00 (12)
C1—C7—H7A	108.8	C4—C5—H5	120.0
C8—C7—H7B	108.8	C6—C5—H5	120.0
C1—C7—H7B	108.8	C1—C6—C5	121.00 (11)
H7A—C7—H7B	107.7	C1—C6—H6	119.5
C2—C3—C4	120.94 (12)	C5—C6—H6	119.5
C2—C3—H3	119.5	C5—C4—C3	119.53 (12)
C4—C3—H3	119.5	C5—C4—H4	120.2
N1—C8—C7	179.54 (14)	C3—C4—H4	120.2
			
C6—C1—C2—C3	0.04 (16)	C3—C2—C9—C10	103.33 (13)
C7—C1—C2—C3	177.52 (11)	C1—C2—C9—C10	−77.54 (13)
C6—C1—C2—C9	−179.09 (11)	C2—C1—C6—C5	0.00 (17)
C7—C1—C2—C9	−1.61 (15)	C7—C1—C6—C5	−177.43 (10)
C6—C1—C7—C8	−5.75 (16)	C4—C5—C6—C1	0.15 (18)
C2—C1—C7—C8	176.84 (10)	C6—C5—C4—C3	−0.34 (18)
C1—C2—C3—C4	−0.23 (17)	C2—C3—C4—C5	0.38 (18)
C9—C2—C3—C4	178.91 (11)		

### Hydrogen-bond geometry (Å, °)

**Table:**

*D*—H···*A*	*D*—H	H···*A*	*D*···*A*	*D*—H···*A*
C9—H9A···N1^i^	0.99	2.57	3.5605 (18)	176
C9—H9B···N1^ii^	0.99	2.56	3.5210 (17)	165

Symmetry codes: (i) −*x*+3/2, *y*+1/2, −*z*+3/2; (ii) *x*−1/2, −*y*+1/2, *z*+1/2.
